# Optical Inspection and Morphological Analysis of *Diospyros kaki* Plant Leaves for the Detection of Circular Leaf Spot Disease

**DOI:** 10.3390/s16081282

**Published:** 2016-08-12

**Authors:** Ruchire Eranga Wijesinghe, Seung-Yeol Lee, Pilun Kim, Hee-Young Jung, Mansik Jeon, Jeehyun Kim

**Affiliations:** 1 School of Electronics Engineering, College of IT Engineering, Kyungpook National University, 80, Daehak-ro, Buk-gu, Daegu 41566, Korea; eranga@knu.ac.kr (R.E.W.); jeehk@knu.ac.kr (J.K.); 2 School of Applied Biosciences, Kyungpook National University, 80, Daehak-ro, Buk-gu, Daegu 41566, Korea; leesy1985@gmail.com; 3 OZ Tec, No. 901, IT Convergence Industrial building, 47, Gyeongdae-ro, 17-gil, Buk-gu, Daegu 41566, Korea; pukim@oz-tec.com

**Keywords:** optical coherence tomography, SS-OCT, circular leaf spot (CLS) disease, *Diospyros kaki*, persimmon leaf

## Abstract

The feasibility of using the bio-photonic imaging technique to assess symptoms of circular leaf spot (CLS) disease in *Diospyros kaki* (persimmon) leaf samples was investigated. Leaf samples were selected from persimmon plantations and were categorized into three groups: healthy leaf samples, infected leaf samples, and healthy-looking leaf samples from infected trees. Visually non-identifiable reduction of the palisade parenchyma cell layer thickness is the main initial symptom, which occurs at the initial stage of the disease. Therefore, we established a non-destructive bio-photonic inspection method using a 1310 nm swept source optical coherence tomography (SS-OCT) system. These results confirm that this method is able to identify morphological differences between healthy leaves from infected trees and leaves from healthy and infected trees. In addition, this method has the potential to generate significant cost savings and good control of CLS disease in persimmon fields.

## 1. Introduction

Circular leaf spot (CLS) disease is the most destructive disease in persimmon cultivation, and occurs frequently in Spain, Korea, and Japan [1,2]. CLS is caused by the pathogen *Mycosphaerella nawae* and leads to discoloration and defoliation of leaves starting from the midrib or the middle vein, which is connected to the petiole of the leaf, and the premature abscission of fruits [3]. During disease outbreaks, severe economic losses in persimmon cultivation have a direct impact on farmers [4]. Therefore, CLS should be controlled to maintain persimmon yield. Early detection is essential to controlling the disease since it has a long incubation period (90~120 days) compared to other fungal pathogens [5]. The efficient control of CLS requires rapid and rigorous diagnosis. Currently, inspections for plant physiological disorders and diseases are carried out visually, using costly conventional optical methods such as light microscopy, scanning electron microscopy, transmission electron microscopy, and magnetic resonance imaging (MRI) [6,7]. Due to the high cost and necessity of a destructive sample preparation, cost-effective and non-destructive methods are preferred for plant disease analyses. Although MRI provides high transverse resolution, diagnosis can be difficult in areas where diseased and healthy tissues generate signals of similar intensity. Due to the long incubation period of the CLS disease (90~120 days), accurate identification of the infected leaf samples at the initial stage using polymerase chain reaction (PCR), histological sectioning methods, and manually controlled existing methods is a difficult goal to attain in agriculture [8]. Since both infected and healthy-looking (but infected) samples have similar external characteristics, visual or manual identification is difficult. Moreover, due to the destructiveness, time consumption, and lesser accuracy of the conventional PCR and histological sectioning methods, an optical inspection method such as OCT has various advantages over the existing methods owing to its high-resolution, real-time, and non-destructive imaging capability. Importantly, CLS disease and diagnostic protocols have not been performed using non-destructive optical methods and have therefore not been reported in the literature to date.

To explore the morphological differences in CLS-infected leaf samples at an initial stage, we adapted optical coherence tomography (OCT), which is a non-destructive optical imaging modality that is capable of providing cross-sectional and three-dimensional images [9,10]. Although the transmission depth of OCT is limited, it offers a high level of sensitivity when capturing depth-resolved images with a high signal-to-noise ratio. Thus, OCT is widely applied in various fields such as ophthalmology [11,12], dentistry [13], otolaryngology [14], and inspection of industrial defects [15]. Recently, OCT has become a prominent imaging modality in the field of agriculture. Several research groups have adapted OCT for the microstructural analysis of kiwi and apple peels to introduce three-dimensional image protocols [16,17]. Furthermore, differences in the hull thickness of four different species of lupin seeds were studied using OCT to measure the precise optical thickness of the layers [18]. Numerous studies have been performed to inspect various infections in melon seeds, cucumber seeds, apple leaves, and orchids, which were quantitatively and qualitatively analyzed to limit the spread of diseases in the plantations [19,20,21,22]. Several reports on the applicability of OCT to plant tissues have been published, which describe the detection of defects, rot, and disease in onions [23,24].

In this pilot study, we describe an optical inspection process used to analyze morphological variations of persimmon leaves, which are caused by *M. nawae*. Leaf samples were collected from healthy trees, infected trees, and healthy-looking leaves from infected trees (leaves on infected trees that appear healthy). The main objective of this study was to introduce a CLS disease inspection protocol by characterizing the reduction of the palisade parenchyma cell layer thickness in the leaf cross-section to limit the spread of the disease. Finally, we performed a histopathological study to further analyze the correlation between OCT images and the biological region of interest. To the best of our knowledge, this is the first demonstration of OCT as an agricultural sensor to detect morphological differences owing to a persimmon-related disease in the field of agriculture.

## 2. Materials and Methods

### 2.1. Preparation of Plant Leaf Samples

All the persimmon leaf samples used in this study were collected from persimmon orchards in Korea. Image acquisition was carried out within 2 h of sample collection in order to minimize tissue damage, and all the collected samples were kept inside a particular ice box during the entire experiment to overcome the drawback of dehydration. Leaf samples were collected from healthy and CLS-infected trees and in addition, leaf samples that appeared healthy were collected from infected trees to investigate morphological variations. All three leaf categories were imaged using high-resolution, real-time, and non-destructive swept source optical coherence tomography (SS-OCT) imaging method which has various advantages over existing methods such as Polymerase Chain Reaction (PCR) and histological sectioning methods [8].

Also, the samples subsequently underwent histological analysis. To observe the internal structures of leaves, samples were fixed in 2% paraformaldehyde and 2.5% glutaraldehyde in 0.05 M sodium cacodylate buffer for 24 h under vacuum conditions. Next, the samples were washed in distilled water and dehydrated in a graded ethanol series—30%, 50%, 70%, 80%, and 90%, absolute ethanol—for 20 min. The dehydrated samples were infiltrated with propylene oxide and embedded in Spurr’s resin. Later, the samples were sectioned with an Ultra microtome (MT-7000, RMC, Tucson, AZ, USA). The sections were stained with 2% methylene blue and observed under a light microscope (BX50, Olympus, Tokyo, Japan).

### 2.2. SS-OCT Experimental System

**Figure 1:**
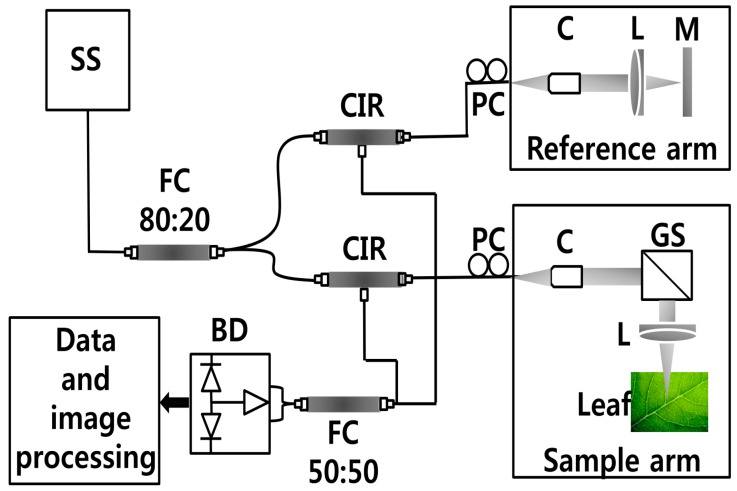
SS-OCT system configuration. Abbreviations: BD—balanced detector, C—collimator, CIR—circulator, FC—fiber coupler, GS—galvano-scanner, L—lens, M—mirror, PC—polarization controller, SS—swept source laser 1310 nm.

In this study, samples were imaged using a compact SS-OCT system (Oz-tec., Gyeonggi-do, Korea) operating at 1310 nm. Figure 1 depicts the system configuration of the established SS-OCT system. The assembled system employs a commercially available 50 kHz wavelength-swept semiconductor laser (Axsun Technology, Billerica, MA, USA) with a sweep bandwidth of 110 nm and average output power of 20 mW. Additionally, it comprised an AC-coupled dual balanced receiver with a 1 GHz bandwidth (PDB481C-AC, Thorlabs Inc., Newton, NJ, USA), which was used as the detector. Digitization of the OCT signal was achieved using a 12 bit 1.8 GS/s digitizer (ATS9360, Alazar Technologies Inc., Pointe-Claire, QC, Canada). Graphics processing unit (GPU)-based software was used to control the hardware of the imaging modality. The axial resolution of the system is 7.8 μm in air and the transverse resolution of the system is 16 μm in air. The developed OCT system was implemented to diagnose the physical state of the leaf samples, and 50 leaf samples from each leaf category were collected for the experiment. All the leaf samples were scanned with a sufficient cross-sectional scanning range of 3 mm since the diameter of the circular spot was measured to be less than 2.5 mm after the incubation period (120 days). According to the agricultural protocol of the CLS disease, the leaf region of interest was midrib or the middle vein, which is connected to the petiole of the leaf. Therefore, to increase the accuracy of the experiment, six 2D OCT images were acquired from 6 positions near the midrib or middle vein were diagnosed in each leaf prior to the occurrence of leaf spots. However, due to the similarity of the obtained images, 2D OCT image of a single representative position from each leaf category is shown in the results.

## 3. Results

### 3.1. Two-Dimensional Morphological Inspection

**Figure 2:**
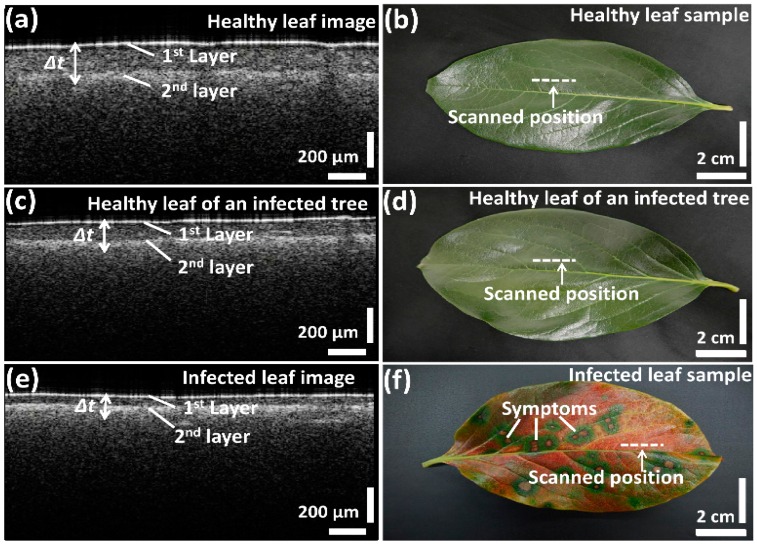
Two-dimensional SS-OCT images and photographs of the leaf samples; (**a**,**b**) healthy leaf; (**c**,**d**) healthy leaf of an infected tree; (**e**,**f**) infected leaf.

We first compared the OCT images of leaf samples from all three categories (healthy, infected, and healthy-looking leaves from an infected tree). To confirm the consistency of the screening over various leaf samples, scanning was performed closed to the midrib or the middle vein region, which is connected to the petiole of the leaf. Figure 2 depicts the representative two-dimensional (2D) OCT images along with the photographs of the leaf samples. The photographs emphasize the distinguishable topographical (leaf top surface) differences between infected and healthy leaf samples. However, no topographical difference can be observed between healthy leaves and healthy-looking leaves from an infected tree. Cross-sectional images (Figure 2a,c,e) revealed a clear difference in the palisade parenchyma layer thickness. Although the physical appearance of the leaves in the photographs looks similar, the OCT cross-sectional images of healthy leaves from healthy trees and healthy leaves from infected trees show notable morphological differences. The 2D OCT images revealed the reduced palisade parenchyma layer thickness, which is the initial symptom of CLS disease in the leaves from infected trees having the physical appearance of healthy leaves. Thus, these results suggest that OCT characterization can be potentially applied to investigate the symptoms of CLS at an initial stage.

### 3.2. CLS Disease Inspection

**Figure 3:**
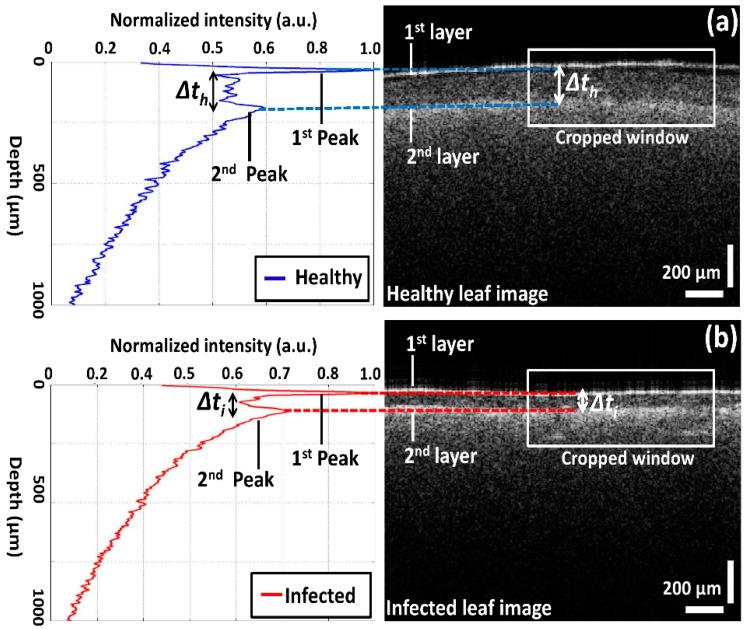
A-scan profiles of healthy and infected leaves and corresponding layer information: (**a**) healthy leaf; (**b**) infected leaf. White square region depicts the selected cropped window for averaging 200 intensity signals.

**Figure 4:**
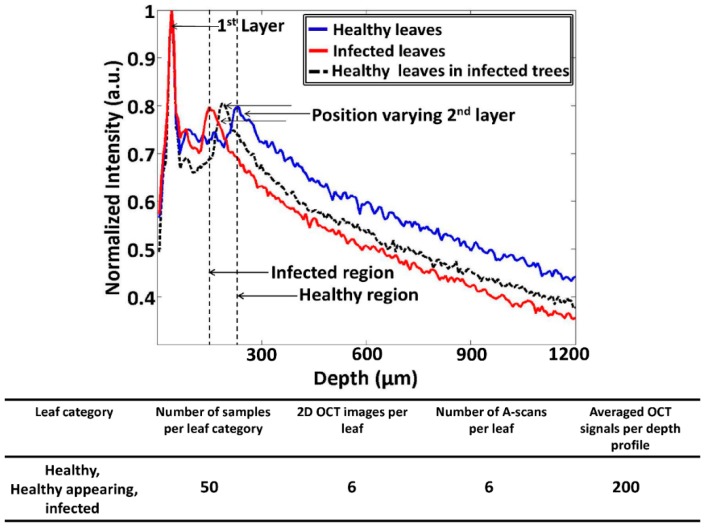
The A-scan profile results comparison for healthy, infected, and healthy-looking leaves from infected trees. The table depicts all the experimental factors related to the A-scan profile analysis.

Due to the multi-layered structure and variation in the absorption coefficient, it is difficult to analyze precise differences in thickness between all leaf layers using OCT images. Nevertheless, the thickness between two layers can be identified through the corresponding distance between OCT signals–based A-scan profile (amplitude scan) peaks, which was obtained from the 2D OCT image of a single representative position of each leaf category. For the A-scan profile analysis, a Matlab (Mathworks, Natick, MA, USA) software–based program was coded to search the intensity peaks in the depth direction. During the A-scan process, a 2D OCT image was loaded and a peak search algorithm-based cropped window with 200 intensity signals (A-scan lines) was applied. The peak search algorithm detects the maximum intensity in the individual A-scan line sequentially. Then all the peak positions in all 200 A-scan lines were rearranged while matching the peak intensity index in the A-scans to flatten the image. Due to the physical structure of the leaf, the 2D OCT cross-sectional image is an unflattened image containing the maximum intensity index positions at different positions. Therefore, the index positions with high intensity should be rearranged and matched linearly to obtain a flattened image. Then, all the rearranged and flattened A-scan lines were summed up and averaged to obtain a single averaged A-scan profile as shown in Figure 3 and Figure 4. To obtain a stable intensity profile, the A-scan intensities were normalized by dividing them into the maximum values. Additionally, we implemented a median filter in the software program to compensate for the speckle noise to obtain a clear intensity plot without any noise destruction [19]. Figure 3 depicts representative A-scan profiles of healthy and infected leaf samples with internal layers localized at different depth levels. For brevity, only the peak OCT signals will be described. The width of the peak information for each A-scan profile corresponds to the two leaf layers, which are the epidermal layer and spongy parenchyma layer in each OCT image, and Δ*t* represents the thickness of the palisade parenchyma layer between the aforementioned two layers. The distance (Δ*t)* between the two main A-scan profile peaks was calculated to acquire the thickness of the palisade parenchyma cell region. The refractive index of each leaf component is unique and different from the others. Also, the differences of the refractive index play an important role in obtaining scattering information from the leaf internal layers. Even though each component of the leaf tissue has a unique refractive index, we implemented 1.42, which is the refractive index of plant cells. A distinguishable variation in thickness can be recognized in the A-scan profile of the infected leaf compared to that of the healthy leaf.

Here, the Δ*t* magnitude seen in Figure 3b was reduced and moved towards the first peak as a result of CLS disease. The aforementioned reduction in thickness (Δ*t)* is considered to be the main initial symptom of the disease, and it is caused due to the reduction of the palisade parenchyma cell layer. Hence, it is essential to identify the palisade parenchyma cell layer thickness at an earlier disease stage in order to determine the physical status of the leaf sample. The A-scan profile results comparison for healthy, infected, and healthy-looking leaves from infected trees illustrating the representative OCT signal information behavior of each leaf category is shown in Figure 4. Additionally, a table containing all the experimental and numerical factors is shown along with Figure 4. The healthy and infected peak information (width of the peak) indicated in the A-scan profiles are consistent with the previous results. However, the width of the peak for healthy-looking leaves from infected trees was located between the healthy and infected peaks. Therefore, OCT is capable of identifying the physical state of the leaves at an initial stage of CLS infection.

### 3.3. Histological Analysis

**Figure 5:**
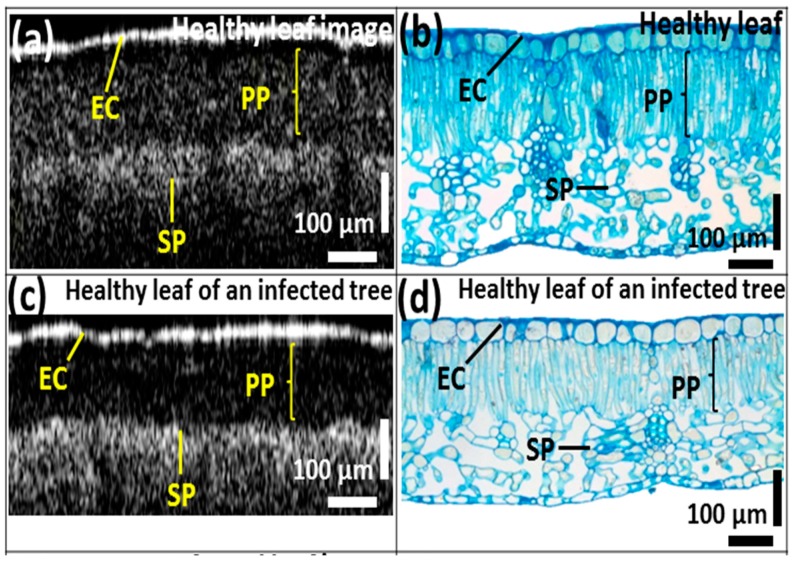

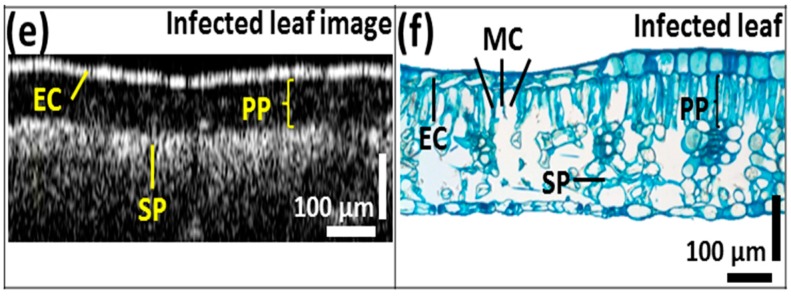
Comparison between (**a**,**c**,**e**) OCT images; (**b**,**d**,**f**) histological sectioning images of three leaf categories. EC—epidermal cells, PP—palisade parenchyma, SP—spongy parenchyma, MC—mycelium cells.

We compared the OCT images of leaves from all three categories with those of images of histological sections stained with methylene blue. In Figure 5, the epidermal cell layer and palisade parenchyma cell layer of the acquired OCT images were closely correlated with the histological images showing distinctive leaf morphological boundaries. However, the plant cells of the spongy parenchyma are barely distinguishable due to the depth-dependent sensitivity loss of the OCT system. In the OCT images, the thickness of the palisade parenchyma cell layer in healthy samples was about 202.81 ± 1.01 μm. Furthermore, a reduction in the aforementioned cell layer thickness was identified in healthy leaves collected from infected trees (appearing healthy), which was about 147.88 ± 0.74 μm, and that of CLS-infected leaves was about 84.51 ± 0.42 μm compared to healthy leaves (Figure 5b,d,f). This reduction in thickness can also be visualized through the histological sectioned images. In addition, the OCT signal reduced with the increasing depth of the leaf sample.

### 3.4. Average Thickness Evaluation of Leaf Cross-Sectional Layers

**Figure 6:**
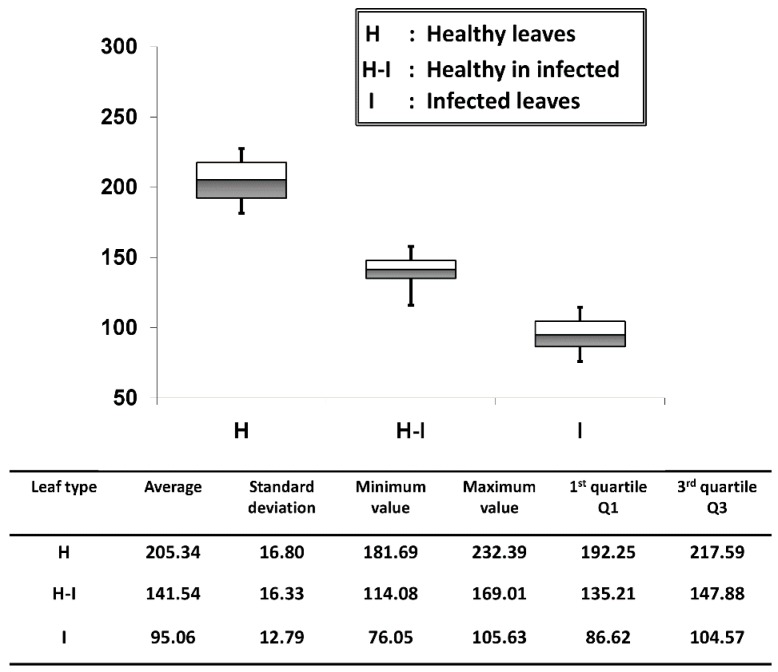
Average palisade parenchyma layer thickness fluctuation of each leaf category. The table depicts all the statistical values and quantifications related to the graph.

We evaluated the palisade parenchyma layer thickness of all six positions per leaf sample and for all 50 samples per each leaf category (50 leaf samples were tested from each leaf category) and averaged them. The thickness quantification was obtained through the OCT A-scan profile signals and this was achieved through an automated signal processing program [19]. On the OCT images, the layer thickness of the palisade parenchyma cell layer in each leaf category was quantified and the results are shown in Figure 6. The average palisade parenchyma layer thickness of the healthy leaf category was about 205.34 ± 16.80 μm e bars indicate ± standard deviation). A considerable reduction in thickness was observed in healthy-looking leaves from infected trees, and the average thickness was about 141.54 ± 16.33 μm. The average thickness identified in infected leaf samples was about 95.06 ± 12.79 μm, which shows the lowest thickness range among all three categories. As a consequence, the quartile region (Q1~Q3) of the results can be implemented as a thickness threshold range to obtain rapid confirmation about the physical state of the leaf by only measuring the thickness difference between the first two peaks of the A-scan profiles, which represent the epidermal and spongy parenchyma cell layers. The statistical values and quantifications such as the average, standard deviations and errors, and minimum and maximum fluctuation range were included in a summarized table along with the graph. Therefore, the efficacy of the proposed method can be enhanced by analyzing the physical state of the leaf at an initial stage using the performed precise quantification method.

## 4. Discussion and Conclusions

Accurate morphological and quantitative information has advantages in agriculture for diagnostic evaluations of plant diseases. Hence, we investigated the capability of OCT to diagnose CLS disease caused by the fungal pathogen *Mycosphaerella nawae* in persimmon leaves. The inspection was carried out using three categories of leaves—healthy and infected leaves and healthy-looking leaves from infected trees. The internal leaf layers were visualized using OCT images. It is of note that the precise thickness of the palisade parenchyma layer was analyzed to investigate the thickness variation according to the corresponding leaf category. The results confirmed there were clear differences in thickness between healthy and infected leaf samples, which could not be identified using conventional methods (Figure 2 and Figure 3).

Previous reports on the typical symptoms of *M. blotch*–infected apple leaves have found differences in leaf thickness between healthy and infected apple leaves [21]. Moreover, low exposure to ozone can reduce the thickness of the birch tree (*Betula pendula* Roth). As a result, the observed difference in the cross-sectional layer thickness between healthy leaves, healthy leaves from infected trees, and infected leaves can be explained based on findings from previous reports. Furthermore, the A-scan profiles shown in Figure 3 quantitatively verify the variation in thickness. Subsequently, a distinguishable variation in thickness compared to that in healthy and infected leaves could be accurately identified in leaf samples that appeared healthy but were collected from infected trees, thus favoring diagnosis and the prevention of disease spread at an initial stage. These findings indicate that the OCT method has the ability to diagnose CLS disease based on the analysis of cross-sectional thickness, which is impossible to distinguish with the naked eye. Furthermore, the proposed method may be used to control the spread of this disease in the persimmon industry. Of note, the genus *Mycosphaerella* causes foliage disease in multiple hosts. Therefore, the proposed disease inspection protocol and the OCT system can be implemented as a suitable agricultural sensor to diagnose other diseases caused by the genus *Mycosphaerella*, and the implementation of OCT is suitable for the rigorous, non-destructive, and rapid diagnosis of CLS disease in the persimmon industry.
